# The physics of sociality: Investigating patterns of social resource distribution among the *Pan* species

**DOI:** 10.1016/j.isci.2025.113507

**Published:** 2025-10-24

**Authors:** Edwin J.C. van Leeuwen, Diego Escribano, Zanna Clay, Marcel Eens, Jean-Pascal Guéry, Daniel B.M. Haun, Stephanie Kordon, Suska Nolte, Nicky Staes, Jeroen M.G. Stevens, Jonas Torfs, José A. Cuesta, Angel Sánchez

**Affiliations:** 1 Animal Behavior and Cognition, Department of Biology, Utrecht University, Padualaan 8, 3584 CA Utrecht, the Netherlands; 2 Department for Comparative Cultural Psychology, Max Planck Institute for Evolutionary Anthropology, Deutscher Platz 6, 04103 Leipzig, Germany; 3 Universidad Carlos III de Madrid, Departamento de Matemáticas, Grupo Interdisciplinar de Sistemas Complejos (GISC), 28911 Leganés, Spain; 4 Department of Psychology, Durham University, South Road, Durham DH1 3LE, UK; 5 Behavioral Ecology and Ecophysiology Group, Department of Biology, University of Antwerp, Universiteitsplein 1, 2610 Wilrijk, Belgium; 6 Zoological Park La Vallée des Singes, Romagne, France; 7 Centre for Research and Conservation, Royal Zoological Society of Antwerp, Koningin Astridplein 26, 2018 Antwerp, Belgium; 8 SALTO Agro- and Biotechnology, Odisee University of Applied Sciences, Hospitaalstraat 23, 9100 Sint Niklaas, Belgium; 9 Instituto de Biocomputación y Física de Sistemas Complejos (BIFI), Universidad de Zaragoza, 50018 Zaragoza, Spain

**Keywords:** Biological sciences, Zoology, Evolutionary biology

## Abstract

Humans invest social resources in predictable “circles of friends,” from intimate ties to acquaintances. Whether these concentric social bonding dynamics (CSBD) are uniquely human or shared with other primates is unclear. We applied the CSBD model to 24 groups of bonobos and chimpanzees (*N* = 284) to test for evolutionary continuity and potential age-related changes in social selectivity. Results show that both ape species distribute social resources in ways resembling humans, supporting shared socio-cognitive structuring within the hominoid lineage. Yet clear species differences emerged: bonobos distributed resources more evenly across group members, while chimpanzees concentrated them more selectively. Moreover, chimpanzees—but not bonobos—became increasingly selective with age, mirroring human patterns of intensified interactions with fewer partners in later life. These findings suggest that our closest living relatives structure their social networks in ways that parallel humans', while also pursuing species-specific strategies that reveal both shared evolutionary roots and divergent pathways of primate sociality.

## Highlights

• Bonobos and chimpanzees show human-like concentric social bonding dynamics
• Larger ego-network sizes correlate with higher skew in social resource distribution
• Bonobos distribute social resources more evenly than chimpanzees
• Older chimpanzees focus their grooming efforts evenly on fewer social partners

## Graphical abstract

**Figure:**
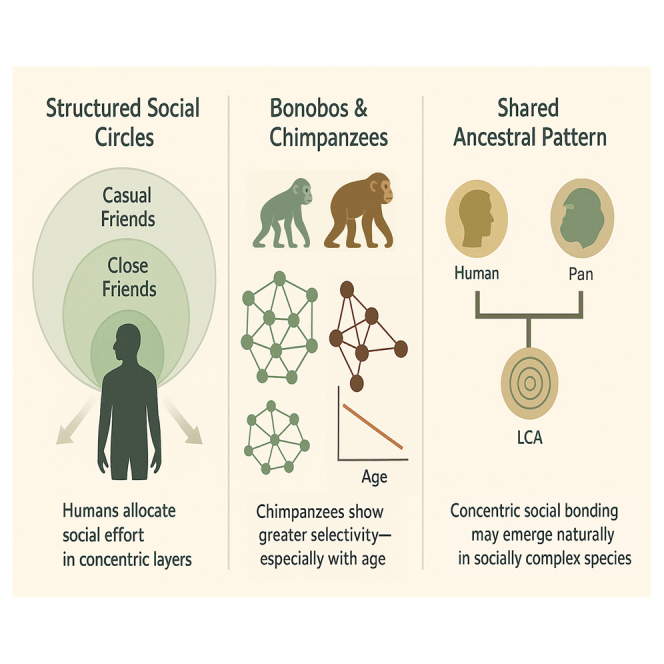

## Introduction

Human relationships are structured hierarchically,^1^ with layers ranging from intimate relationships to acquaintances. This layered structure can be modeled as resulting from limitations of our social resources.^2^ Our cognitive abilities, such as memory, or attention, and our capacity to respond emotionally to our social environment, have finite limits. Thus, it is pertinent to allocate these limited social resources effectively across relationships, especially considering the fitness advantages such social bonds confer.^3^^,^^4^ Intimate relationships, such as close family members and romantic partners, involve deep emotional connections, shared experiences, and a higher degree of interdependence. Given their importance in our lives, intimate relationships receive a significant allocation of our cognitive resources. As we move down the layers, we encounter relationships of progressively decreasing emotional and cognitive intensity. These include close friends, extended family members, colleagues, and acquaintances. While these relationships still hold significance, they generally require fewer cognitive resources and emotional investment compared to intimate relationships. We can maintain a larger number of such relationships due to their relatively lower cognitive demands. While the hierarchical structure of human relationships is mathematically proven^2^^,^^5^ and quite well-documented, it remains an open question whether other species exhibit similar relationship structures based on such a (social) resource model. Finding similarly layered relationship structures in non-human animals (henceforth “animals”) would have significant implications for our understanding of social complexity and the evolution of social behavior.^6^^,^^7^^,^^8^^,^^9^ It would suggest that the patterns of social resource distributions within socially living animals are driven by general principles of physics rather than a unique feature of the human species. Such findings could provide valuable insights into the relationship between cognitive abilities and social dynamics across species, highlighting shared principles underlying social organization across different taxa.

The social structure – the pattern by which individuals organize themselves in a group^9^ – of relatively large-brained species, such as primates, elephants, dolphins, and certain bird species, shows complex interactions and relationships.^10^^,^^11^^,^^12^^,^^13^ These relationships have been the focus of concerted scientific efforts to unravel the structure of animal societies,^14^ and elucidate under what ecological or cognitive pressures these relationships, also known as social bonds,^15^ emerge and stabilize. In non-human primates (henceforth “primates”), for instance, social bonds are defined as differentiated stable affiliative relationships and are expected to arise for males in contexts where male monopolization is moderate to low, enabling allies to collaborate effectively in within-group political coalitions.^16^^,^^17^ Conversely, female social bonds are believed to stem from the advantages of cooperating with relatives, particularly in socioecological conditions that favor nepotistic behaviors.^18^^,^^19^ Increasingly, it is recognized that these social bonds confer significant benefits to primates.^15^^,^^20^ For example, in several species, these bonds contribute to cohesion and stability, ensuring that individuals reap the benefits of social living, such as protection against predators, access to resources, and reduced physiological stress.^21^^,^^22^^,^^23^ Beyond immediate survival, social bonds can impact long-term outcomes, including reproductive success and longevity.^4^^,^^24^^,^^25^ Primates, in particular, exhibit a high reliance on social bonds due to their complex social structures and reliance on group living.^9^^,^^22^^,^^26^ Yet, an outstanding question remains just exactly how structured their sociality is and how this structure comes about.^8^^,^^27^

With respect to social structuring, we know that primates live in a wide variety of social systems, ranging from stable, cohesive groups to dynamic, fluid networks. Some species, such as chimpanzees and bonobos, exhibit fission-fusion dynamics, where group members split into smaller subgroups and rejoin over time, leading to flexible and constantly shifting social interactions.^28^^,^^29^ Others, such as hamadryas and gelada baboons, form multilevel societies with nested layers of social organization—such as one-male units embedded within larger clans or bands—allowing for both stability and broader social integration.^13^^,^^30^

These varying structures shape the opportunities individuals have to form and maintain social bonds, which in turn influence their access to resources, mating opportunities, and cooperative partners. However, the question remains: how do group-living animals distribute their social resources across their group members? Understanding such energy distributions is key to uncovering the individual-level strategies animals employ within their intricate networks—strategies that ultimately underlie social behavior and affect fitness-related decisions such as alliance formation, cooperation, and conflict mediation.

Here, we test the hypothesis that due to inherent constraints on time and cognition, primates, such as humans, instigate and maintain social bonds following principles of entropy, causing their sociality to be structured in circles of decreasing intensity. To determine whether primates possess a layered relationship structure akin to humans, we model them premised on the following question: given a finite resource, how is energy canalized and distributed across group members? This entropy flux can be understood as a mechanism by which social structure (e.g., multi-level societies) emerges, or at least as a mediating factor in the parameter space defined by genetics, ecology, and behavioral flexibility.^9^^,^^31^^,^^32^ The field that focuses on the applicability of laws of physics to the structuring of social entities is referred to as “social physics.”^33^^,^^34^

Recently, we showed that semi-wild living groups of chimpanzees organize their social lives in a similar way as humans do.^35^ In that study, we focused on chimpanzees’ main social currency, namely allo-grooming. Allo-grooming refers to the behavior where individuals affiliatively interact with each other, typically using their hands or specialized grooming tools such as twigs or leaves, to remove parasites, dirt, and debris from each other’s fur or skin. This behavior serves several important functions in primate social groups, including social bonding and recruitment for future support.^36^^,^^37^^,^^38^^,^^39^ We found that individuals differed in the extent to which they allocated their grooming efforts across partners (e.g., spreading their grooming thinly across many group members or skewing them toward one or two individuals), but that overall, their distribution patterns matched human interaction patterns in terms of social differentiation.^35^^,^^40^^,^^41^ Moreover, we found the same relationship between grooming differentiation and group size as has been shown in human studies: With larger groups, the tendency for skewing one’s social capital toward a few individuals was more pronounced.^35^ This finding indicates that the human case is not unique, but that there may be a more general principle guiding the patterning of social relationships in social animals. To test the conjecture toward the principle being universal, more animal species and more groups within those species, need to be tested on their within-group social resource distributions.^42^^,^^43^^,^^44^^,^^45^

We analyze data from fifteen groups of bonobos (*Pan paniscus*) and nine groups of chimpanzees (*Pan troglodytes*) with the following three aims: (1) assess whether our closest living relatives distribute their social resources across group members in a similar way as humans do, (2) compare the resource distributions of the two *Pan* species to further investigate attributes of their social systems, and (3) identify socio-demographic determinants of their respective social resource distribution strategies. We focused on allo-grooming as this behavior represents a valuable social commodity in most primate societies.^36^^,^^37^^,^^46^ Furthermore, we chose to focus on bonobos and chimpanzees as both species are closely related to humans,^47^ yet may substantially differ from each other in their social resource distributions, given that bonobos are sometimes regarded as possessing a more egalitarian social structure than chimpanzees (^29^^,^^48^^,^^49^^,^^50^^,^^51^^,^^52^, but see^53^^,^^54^^,^^55^^,^^56^). Also, contrary to chimpanzees, bonobo societies are female-dominated,^29^^,^^50^ which may alter the strategic employment of social resources mediated by sex. Here, we note that we tested zoo- and sanctuary-housed *Pan* apes, which inevitably comes with restrictions in terms of space use opportunities and differences in terms of behavioral determinants such as predation pressures and food availability. Yet, this difference from wild settings does not impede the apes’ opportunity and proclivity to associate with group members selectively, as has been substantially evidenced by studies in (semi-)captive settings.^57^^,^^58^^,^^59^ As such, we may plausibly tap into the apes’ social strategies, and thus validly investigate their social resource distributions. Finally, we investigate the distribution strategies of individuals in both *Pan* species across their age ranges. Humans and other primates have previously been shown to become more socially selective with age.^60^^,^^61^^,^^62^^,^^63^ Overall, we hypothesize that (1) both *Pan* species exhibit similar concentric social bonding dynamics (CSBD) as humans, (2) compared to chimpanzees, bonobos’ CSBD is characteristic of a more egalitarian social structure (i.e., more equal distribution of grooming efforts across group members than chimpanzees),^29^^,^^64^ (3) apes in larger groups have different CSBDs than in smaller groups,^35^^,^^62^ and (4) CSBDs indicate higher social selectivity for older compared to younger individuals, at least for chimpanzees^63^ (*cf*. similar trends in bonobos^62^).

## Results

In this study, we use the η parameter to quantify CSBD—the distribution of social effort across relationships of varying emotional intensity. η is computed by solving an implicit equation that links it to the average relational cost σ, derived from the empirical distribution of relationship weights and total number of ties (see Section 4.2.1). Once estimated, η determines the cumulative fraction of ties up to a given emotional distance, allowing the precise modeling of an individual’s social layering. Positive values of η produce the typical hierarchical structure of expanding, lower-intensity layers, while negative values predict an inverse regime dominated by strong ties. η thus offers a continuous, mechanistic measure of ego-network stratification rooted in cognitive and energetic constraints.

To ensure robust analysis of social resource distributions across group members, we only considered groups with at least 6 individuals (number of groups *N* = 24; chimpanzees *N* = 9; bonobos *N* = 15) and individuals who had groomed at least 5 group members (see also^35^ for a detailed discussion of the data selection process).

### General patterns of concentric social bonding dynamics

**Figure 1:**
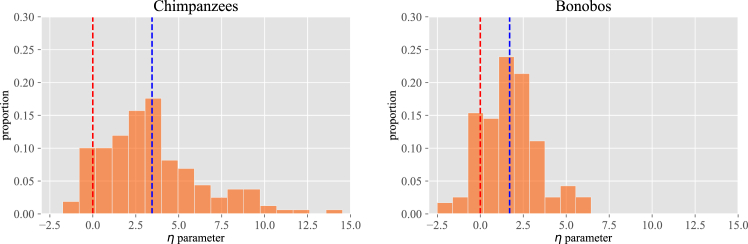
Histogram of η values (*x*-axis) for chimpanzees (left, *n* = 143 individuals) and bonobos (right, *n* = 141 individuals) The red dashed line indicates the change of regime, from negative values of η to positive ones. Negative values for η correspond to inverted structures, with almost all connections being part of the first circle and only a few in the remaining ones. Blue dashed lines indicate the mean value for each of the histograms. Frequency (*y* axis) is expressed in the proportion of individuals.

The ego-networks of the apes (i.e., their individual network structures) can be well described by a parameter η which, if positive, indicates a typical CSBD distribution (see Section 4.2.1), with a few animals among those that are the most groomed and a larger number of animals in a second group that received less grooming (analogous to the ideas of “best friends” and “friends”). Figure 1 presents histograms for the η values obtained for the two species. We observe that the mean value (blue dashed line) of the histogram is at values similar in magnitude to what has been found for humans.^5^ At the same time, we find that η values tend to be smaller and more aggregated for bonobos than for chimpanzees, indicating a less pronounced circle structure for this species. In other words, bonobos seem to distribute their grooming time in a more uniform manner, while chimpanzees have differentiated groups of individuals in terms of the amount of grooming they give them.

### Species comparisons and demographic influences on concentric social bonding dynamics

After establishing the layered structure of the *Pan* ego-networks and the corresponding parameter η as an appropriate summary variable, we address the dependence of the parameter η on different variables, namely: species, ego-network size, age, sex, group size, and habitat type (sanctuary or zoo-housed), including the interaction between species and age, and species and sex.

#### Global analysis

**Figure 2:**
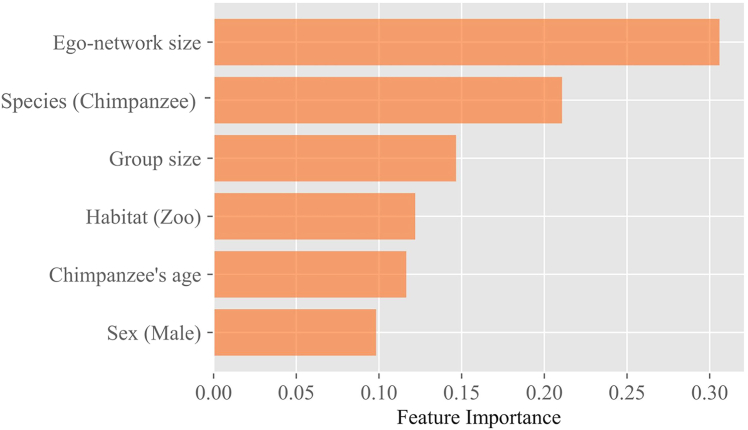
Importance (*x*-axis) of the different features (*y* axis) influencing the parameter η according to the XGBoost model results

To understand the parameters determining η, we used a gradient boosting approach, specifically XGBoost, which iteratively builds decision tree models to minimize prediction errors. By sequentially training each model to correct the residuals of the previous one, XGBoost captures complex, non-linear relationships between features and η with high accuracy and efficiency, and determines feature importance while controlling for the other independent variables (see Section 4.2.2). Using this XGBoost gradient boosting technique, we find that the most important feature affecting η is the apes’ *ego-network size* (Figure 2).

Individuals with large networks of social connections (ego-networks) distribute their social resources among all of them, leading to a more structured organization and a larger value of η. Overall, we observe that the size of ego-networks for apes in zoos is generally smaller than in the sanctuary settings (Figure S1), which we preliminarily attribute to apes in zoo-housed settings having less opportunities for socialization, owing to their typically smaller group sizes (Table S1). The second most important feature is the *species* – a result that coincides with the observation that chimpanzees tend to have larger ego-networks than bonobos (Figure S1), influencing the value of η. *Group size* is the next important variable in agreement with the idea that, when there are less opportunities for social relationships, individuals can devote more resources to each of them, leading to a lower value of η (see^2^). The importance of group size is already less than half that of the ego-network size. Finally, in order of decreasing relevance, we find the type of habitat, the interaction of the species with age (for chimpanzees), and sex to be influencing the value of η (for more details on the direction of the effects, see Section local analysis; Figure 4).

**Figure 3:**
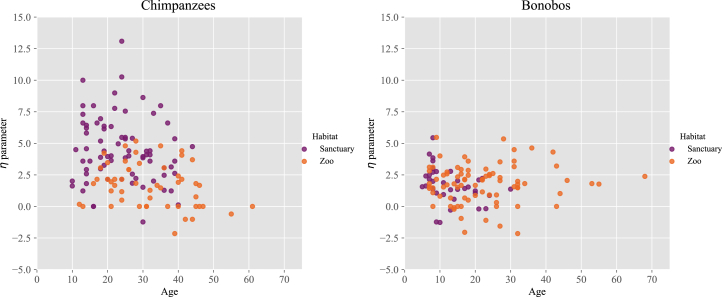
Scatterplot for the magnitude of the η parameter (*y* axis) of chimpanzees (left, *n* = 143) and bonobos (right, *n* = 141) plotted against age in years (*x*-axis) Symbols correspond to different environments/habitats as indicated in the legend.

Confirming our hypothesis, we find evidence that chimpanzees have a lower value of the η parameter with increasing age (Figure 3), which coincides with a decrease in their ego-network sizes (Figure S1). The same pattern is not obviously present in the bonobos, meaning that we do not find evidence that bonobos become more socially selective with age (Figure S2). Moreover, the effect of sex on η was not obviously different for the two species. Notably, the results are controlled for the effect of the other parameters, including group size, which could be suspected of exerting substantial influence across the other effects (see Figures S2–S7 for depictions of all marginal effects using partial dependence plots). Furthermore, we have re-ran the analyses without the largest group of chimpanzees (which may be characterized as an outlier in terms of group size), yet all our results remain the same.

#### Local analysis

**Figure 4:**
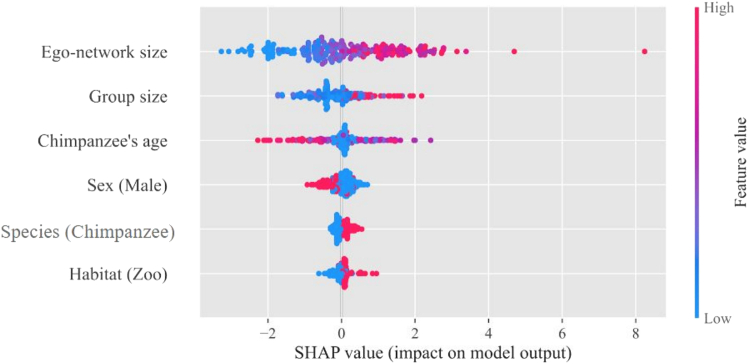
SHAP values arising from the analysis at the individual level according to the XGBoost model results Features are ordered from top to bottom by their overall importance, i.e., features at the top have a greater impact on the model. The color indicates the value of the feature, relating its value distribution with the distribution of SHAP values. Points to the right (or left) of the vertical zero line indicate a positive (or negative) effect on the predicted value of η. The horizontal distance from zero shows the difference of the model’s output when this feature is considered/ignored.

While feature importance provides a global interpretation of the model (i.e., which features are important overall), in what follows, we will use SHAP (Shapley Additive explanations). SHAP decomposes the prediction of a particular data point, detailing the contribution of each feature to that specific prediction.^65^ This method provides a local interpretation, which is crucial for understanding the influence of individual features on particular outcomes within the dataset (see Section 4.2.3). The results of this analysis are presented in Figure 4.

Figure 4 shows the distribution of the effects of each feature across all data points in the dataset. Each point on the plot represents the SHAP value of a feature for a specific instance, allowing us to see how that feature affects the prediction in different situations. Here, it is good to note that the SHAP values represent conditional effects, which means that, regardless of any co-variation between the features (see Table S2), the effects are assessed while controlling for the presence of the other effects.

As can be observed in the figure, *ego-network size* is the most relevant variable for predicting individual characteristics, followed by the *group size*. Both these features have a large positive impact on the value of η. It is important to note here that a larger ego-network or group size does not necessarily equate to a larger η value: individuals in large groups could also distribute their social resources evenly across their group members, but apparently the apes do not, just like humans.

In contrast with the global analysis arising from gradient boosting, SHAP shows that the *interaction of chimpanzees with age* is the third relevant factor, while the three remaining values contribute little to the prediction of the individual η values. Interestingly, SHAP value analysis allows us to identify the direction of the different effects. Thus, we observe that the ego-network has a positive effect on the value of η: the larger the size of the ego-network, the larger the value of η (Figure 4). Group size goes in the same direction. These two findings are once again aligned with the idea that the larger the number of possible grooming partners, the more pronounced the circle structure, with a few partners receiving a lot of attention and many partners receiving a little. The effect of the interaction between chimpanzees and age is negative, indicating that for chimpanzees the value of η decreases with age, meaning that they start to distribute their social resources more evenly, which coincides with a smaller ego-network, thus smaller circle of friends (see Figures S1 and S2). For the remaining factors that have lower influence, the plot shows that male individuals have smaller values of η than females, that chimpanzees have larger values of η than bonobos, and that apes in a zoo have larger η values as compared to the apes in sanctuaries. While this last result may look counterintuitive considering the arguments we have presented earlier, it can be understood by realizing that SHAP values explain differences between variables keeping all others constant. Therefore, our results indicate that, *ceteris paribus*, the value of η would be larger for apes in zoos than for those in the wild. However, that is not the actual case because the dependence on other variables, being more influential, prevents this from occurring.

Finally, we can gain yet deeper insight into the grooming behavior of chimpanzees and bonobos by analyzing the relationship between the η parameter and the network structure, focusing on *modularity* as our magnitude of interest. Modularity represents the difference between the actual proportion of edges within specified groups and the proportion expected by chance.^66^^,^^67^ This value is positive (and never exceeds 1) when the actual number of intra-group edges surpasses what would be anticipated randomly. It serves as an indicator of a network’s organizational level, evaluating the intensity of its segmentation into modules (alternatively known as groups, clusters, or communities). Networks exhibiting high modularity feature closely knit links among nodes within the same module, yet few connections across nodes belonging to different modules. With the η value representing individuals’ social resource distributions, we may expect an impact of (or effect from) the group’s modularity.

**Figure 5:**
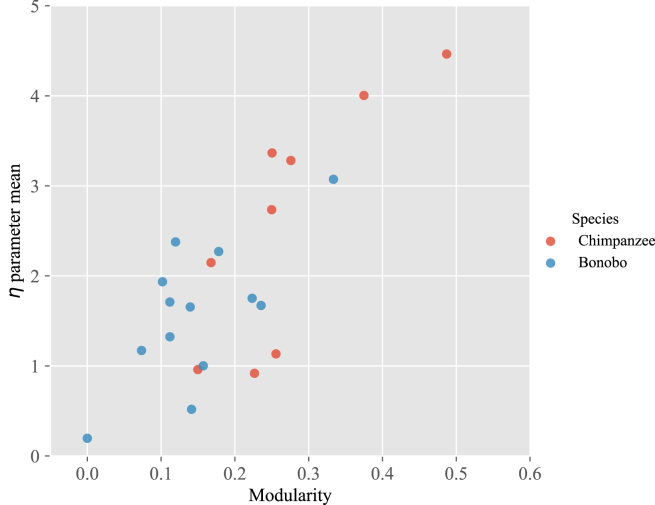
Scatterplot of the mean values of η plotted against the modularity for each group in the sample Data are plotted for each species separately (red = chimpanzees; blue = bonobos).

**Figure 6:**
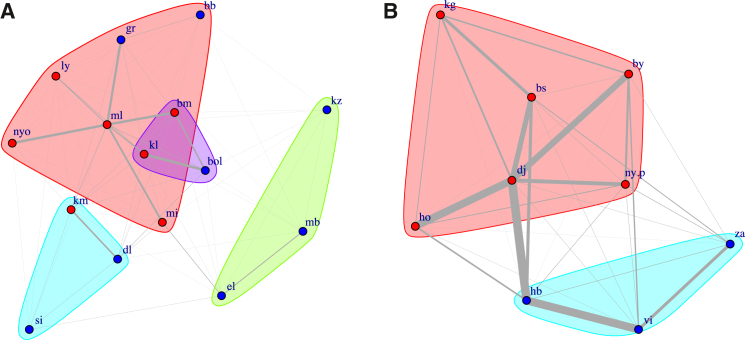
Two grooming networks of sampled bonobos Network (A) has a relatively high modularity Q = 0.17, while network (B) a relatively low modularity Q = 0.07. The colored groupings indicate the number of communities found by a combination of heuristic algorithms.^68^

Our main result can be seen in Figure 5, where we observe clearly that higher values of the average η parameter indicate higher average modularity, i.e., more structure within the network. As an example of the difference between networks with low or high modularity, Figure 6 represents two grooming networks, showing that the one with high modularity can be decomposed into four groups or communities, whereas the one with low modularity has only two groups, and is not clearly differentiated. Thus, given that η is positively correlated with modularity, we find more group structure due to resource distributions characterized by high η values (e.g., more structured groups, on average, in chimpanzees compared to bonobos; also see Figure 5).

## Discussion

We investigated whether principles of social physics apply across closely related species by comparing the structure of social resource distributions in bonobos and chimpanzees (i.e., the *Pan* species) and relating them to established insights from the human species. Overall, the application of social physics to the study of great ape societies offers a promising avenue for identifying (universal) patterns of social resource distribution, which may have substantial ramifications for fitness-relevant behavior such as social bonding, cooperation and social learning.^8^ By delving into these specific quantitative aspects of social interactions, we can uncover insights that transcend species boundaries, contributing to a more holistic understanding of the fundamental principles guiding social organization and resource allocation in animal communities.

Specifically, we studied the most common and pivotal social currency of the *Pan* species – allo-grooming – to identify how the apes distribute this valuable yet finite resource across their group members. Simply put, the question at stake boils down to the following: given limited resources and the fact that your resource donation yields returns not *directly* but *probabilistically*, do you invest all your energy in one or a few group members, or do you invest less energy per capita but across more group members? In humans, we know that generally people use their time and energy selectively such that close friends (including family) receive most of their social resources, followed by more peripheral circles of friends, distant friends, acquaintances and (almost) strangers.^69^ In a recent study, we showed that humans are not unique in this respect: Semi-wild living groups of chimpanzees distribute their resources across group members in a similar way as humans do.^35^ We corroborate this finding with a much larger sample and additionally show that chimpanzees’ sister species - the bonobo - similarly exhibit this typical “human” structure of social resource distribution. Specifically, they exhibit the same organization in circles, in which the majority of individuals divide their attention into a few individuals who receive a lot of grooming and a larger number of partners to whom they devote considerably less time. A small number of apes show an inverted regime, also found in human subjects, in which they groom a relatively small number of others investing roughly the same amount of time on each one.

Moreover, we identified several factors explaining variation in individuals’ CSBD strategies. Also, such as in humans, the apes’ CSBDs seemed affected by ego-network size (i.e., the number of their grooming partners) and overall group size, such that a higher η parameter was increasingly found with larger ego-network and group sizes. This indicates that the apes choose to distribute their grooming resources in a more structured/differentiated manner when they have many grooming partners available, and they live in larger groups. Chimpanzees tend to groom more selectively, and hence to have a larger value of the η parameter and a network with more structure, than bonobos, which could indicate that bonobos are more egalitarian or indiscriminate in their social bonding dynamics than chimpanzees.^49^^,^^64^ Here, the reasoning would go as follows: If bonobos distribute their social effort more evenly across their group members compared to chimpanzees (i.e., they have a lower η on average), their networks may show less modularity and more cohesion—i.e., fewer tightly defined subgroups—which could be interpreted as a less complex structure in terms of social partitions. We have also observed that the value of η decreases with age in chimpanzees (while sex and type of habitat seem to influence the structure of their grooming network to a much lesser extent). The lower η value with relatively old age coincided with a decrease in ego-network size in older chimpanzees. This indicates that older chimpanzees distribute their social effort more evenly across their contacts within increasingly smaller ego-networks, reflecting a narrowing of their social circles. This pattern aligns with growing evidence from wild primates showing social selectivity with age—a phenomenon well documented in both chimpanzees and macaques. In wild chimpanzees, for instance, older individuals have been observed to prioritize a few strong and stable relationships over a larger number of weak ties.^63^ Similarly, research on barbary macaques has shown that aging individuals reduce the number of social partners, focusing more selectively on important long-term affiliates.^70^

The declining η values in older chimpanzees could reflect a strategic social investment, wherein individuals shift from broad engagement to deeper connections with select partners. This shift likely optimizes emotional security, predictability, and mutual support, echoing patterns observed in humans under the framework of socioemotional selectivity theory.^71^^,^^72^ Moreover, a smaller ego-network may not imply social disengagement, but rather a refinement of social priorities, possibly shaped by cognitive aging, increased risk aversion, or accumulated social experience. These findings contribute to a broader understanding of how aging may influence social structure in long-lived, socially complex species.^70^^,^^71^

We did not find the same pattern of social selectivity with age in bonobos. Unlike chimpanzees and macaques, older bonobos in our study did not show a clear narrowing of social networks or a more selective distribution of social effort compared to younger conspecifics. This may reflect the species’ more egalitarian and tolerant social structure^29^^,^^49^^,^^52^ (cf.^53^^,^^55^^,^^73^), which could reduce the need for older individuals to prioritize a few strong ties. However, bonobo social dynamics vary across groups (e.g.,^55^^,^^74^^,^^75^), and it remains possible that age-related social changes occur in subtler forms or under specific ecological or social conditions. More longitudinal and cross-group studies are needed to assess whether the absence of social selectivity with age is a consistent feature of bonobo sociality (cf.^62^).

### Limitations of the study

Our study is not without limitations, although we wish to emphasize that we have sampled a relatively large number of groups of the two species involving different types of habitats. Yet, our results should be interpreted with some caution, as our sample included only zoo-housed and sanctuary-living groups, which inevitably means that the respective apes have less degrees of freedom with respect to social navigation than their wild counterparts. However, research on captive populations has demonstrated that *Pan* species selectively interact with one another,^57^^,^^58^^,^^59^ which boosts the validity of inferences regarding their preferences and social resource distributions. Moreover, captivity may have the added advantage that behavioral propensities (such as selectively interacting) can be studied in relatively similar socio-ecological contexts that may influence social patterning substantially in the wild (e.g.,^19^). Finally, we have collected data from populations living in two of the largest great ape sanctuaries of the world, which support the idea that even in environments with the possibility for sub-grouping (i.e., fission-fusion dynamics), the apes’ social choices amount to the posited CSBD.

A related discussion point arises regarding whether our findings can be generalized to wild populations. In particular, do captive apes exhibit similar core grooming networks as wild populations, or do environmental factors lead to distinct patterns of social bonding? Wild bonobos and chimpanzees tend to live in larger groups than the sampled groups in this study, and even though they similarly concentrate their grooming efforts *selectively*, leading to network structure,^29^^,^^76^^,^^77^^,^^78^ this aspect may affect their social patterning dynamics. In principle, our data show that with larger group sizes, the distribution of individuals’ grooming efforts across group members changes toward CSBD, but there may be a ceiling effect of group size after which CSBD does not hold, or other forms of social patterning emerge. Also, the inverted regime in small fission-fusion groups may look different in the wild compared to zoo settings, due to anticipated reunion with the larger core group,^77^ or due to location-specific social bonding (i.e., social niche construction^79^). These questions are exciting and warrant an extension of the social physics approach to wild great ape populations, as this would elucidate the universality of the principles underlying CSBD.

Overall, we find support for an evolutionary signature of resource distribution structures by showing that the *Pan* species – humans’ closest living relatives – organize themselves socially in similar patterns as observed in human social networks. This suggests that a more general principle guides the shape of network structures rather than a derived one purportedly responsible for the structure of human societies. As has been shown while applying principles of physics to understanding the form and function of social networks in humans and now also other species, this phenomenon of social patterning seems to arise because, in the end, allocating grooming time (or friendship intensity^74^) is nothing but a problem of distributing a finite resource (time, cognitive capability) among a certain number of recipients. Therefore, the evolution of social animals such as humans and the *Pan* species, leading to a key role of the social support network for the health and fitness of individuals, should obey this principle, and therefore lead to similar structural features as we have demonstrated in this research. Further analysis of data from other species and habitats could provide more evidence for the universality of this organizing principle (e.g.,^80^).

## Resource availability

### Lead contact

Further information and requests for resources should be directed to and will be fulfilled by the Lead Contacts: Dr. Edwin J. C. van Leeuwen (e.j.c.vanleeuwen@uu.nl) and Prof. Angel Sánchez (anxo@math.uc3m.es).

### Materials availability

This study did not generate new unique reagents.

### Data and code availability

All data used in this study are available at a public repository: https://surfdrive.surf.nl/files/index.php/s/8GW4bVwDllgc4EY. The developed code is available upon reasonable request.

## Acknowledgments

We thank Katherine Cronin for the coordination of data collection at Chimfunshi and Heritier Izansone for the data collection at Lola. We thank the participating zoos and care staff for hosting us for behavioral data collection and for supporting this work: Apenheul (Apeldoorn, the Netherlands), Beekse Bergen (Hilvarenbeek, the Netherlands), Burgers Zoo (Arnhem, the Netherlands), La Valleé Des Singes (Romagne, France), Leipzig Zoo (Leipzig, Germany), Ouwehands Dierenpark (Rhenen, the Netherlands), Twycross Zoo (Twycross, United Kingdom), Wilhelma Zoological and Botanical Garden (Stuttgart, Germany), Wuppertal Zoo (Wuppertal, Germany), Zoo Frankfurt (Frankfurt-Am-Main, Germany) and Zoo Planckendael (Mechelen, Belgium). Similarly, we thank the Chimfunshi Wildlife Orphanage staff and caretakers, and the staff and caretakers of Lola ya Bonobo Sanctuary, and the Zambian Wildlife Authority and the Ministries of Research and Environment in the Democratic Republic of Congo for their collaboration and support. We also thank all the students who were involved in data collection: Ilke Fromont (University of Antwerp), Annemieke Podt, Sanne Roelofs, Martina Wildenburg and Sjoerd Beaumont (all Utrecht University). EJCvL was funded by the European Union under ERC Starting Grant no. 101042961 – CULT_ORIGINS and by the Dutch Research Council (NWO) under grant no. VI.Vidi.231G.071. Views and opinions expressed are however those of the author(s) only and do not necessarily reflect those of the European Union or the European Research Council Executive Agency. The following researchers were funded by 10.13039/501100003130Research Foundation Flanders N.S. (grant n◦ 12Q5419N) and J.R.R.T. (grant n◦ 1124921N). The Antwerp Zoo Centre for Research and Conservation is funded by the Flemish government. J.A.C. and A.S. acknowledge support from the PID2022-141802NB-I00 (BASIC) grant funded by MCIN/AEI and by “ERDF A way of making Europe.” A.S. acknowledges financial support from grant “MapCDperNets” -- Programa Fundamentos de la Fundación BBVA 2022.

## Author contributions

EJCVL, DE, JAC, and AS conceived the study; EJCVL, DE, JAC, and AS wrote the article; DE performed the formal analysis; JAC and AS supervised the project; ZC, ME, JPG, DBMH, SK, SN, NS, JMS, and JT provided resources and edited the article.

## Declaration of interests

The authors declare no conflict of interest to exist.

## STAR★Methods

### Key resources table

**Table:** 

REAGENT or RESOURCE	SOURCE	IDENTIFIER
**Deposited data**		

Raw data (and code)	This paper	https://surfdrive.surf.nl/files/index.php/s/8GW4bVwDllgc4EY.

**Experimental models: Organisms/strains**		

Bonobos (Pan paniscus) and chimpanzees (Pan troglodytes)	Zoological and sanctuary settings	N/A

**Software and algorithms**		

XGBoost (gradient boosting framework)	Open Access	https://xgboost.readthedocs.io/en/stable/

**Other**		

Observer XT software	Noldus, the Netherlands	https://noldus.com/observer-xt-animal
ZooMonitor software	Lincoln Park Zoo, United States of America	https://zoomonitor.org/

### Experimental model and study participant details

Here, we provide information on the study groups (*N*=24) and their housing environment. Overall, we tested 284 great apes (*N*_Bonobos_ = 141 across 15 groups (51 males, 90 females); mean age = 20.7 years, age range = 5 – 69 years; *N*_Chimpanzees_ = 143 across 9 groups (51 males, 92 females); mean age = 25.7 years, age range = 6 - 61 years; see Table S1). In addition to zoo- and sanctuary-specific adherence to local stipulations, this study conformed to the ASAB guidelines for animal behavior research.^81^

#### Ethics statement

Animal husbandry and research protocols complied with international standards (the Weatherall report), institutional guidelines (zoos) and national standards for the treatment of animals as stipulated by the local wildlife authorities (Zambian Wildlife Authority and the Ministry of Research and the Ministry of Environment in the Democratic Republic of Congo). The Chimfunshi Research Advisory Board reviews projects for chimpanzee safety and welfare, and functions as an independent entity for evaluating ethical and feasibility criteria for each study proposed to be conducted at Chimfunshi since 2011.

#### African sanctuaries

##### Chimfunshi Wildlife Orphanage Trust (Zambia)

The Chimfunshi Wildlife Orphanage is a sanctuary located in the north-western part of Zambia, close to the border with the Democratic Republic of Congo. At Chimfunshi, the chimpanzees live in large, forested (Miombo) enclosures,^82^ stay outside overnight and only come indoors for supplemental feeding between 11.30h–13.30h (for more details, see e.g.,^83^). For this research project, we focus on data from the 4 social groups at the Project area, all of which have been stable in terms of demography for at least 18 years (Table S1).

##### Lola Ya Bonobo (Democratic Republic of Congo)

Lola Ya Bonobo is a bonobo sanctuary located in the Democratic Republic of Congo. The study site includes three separate enclosures with a total ground area of 30 ha (range enclosure size: 5-15 hectares). All these outside enclosures provide a semi-natural environment with *ad libitum* access to water by means of a lake, floating stream or a pool and are further composed of secondary rainforest, grasslands and partly of swampy areas. Each enclosure furthermore includes an inside sleeping area in which individuals spend the night in voluntarily chosen subgroups. Behavioral observations were conducted at the outside enclosures of Group 1 and Group 2 (see Table S1 for details) by SK and a research assistant between July and September 2019.

#### European zoo-settings

##### Data collection part 1 (2011-2022)

Between 2011-2022, we obtained observational data from nine bonobo groups across 7 different zoological institutes in Europe (Table S1). All bonobos were housed adherent to the guidelines of the EAZA Ex situ Program (EEP). Some institutions were visited multiple times during this period, and some institutions housed multiple groups, such that in total 9 compositionally different groups of bonobos were sampled. A group was considered to be compositionally different when they differed in the presence or absence of at least one individual,^84^ since previous research has shown that the removal from or addition to the network of one individual can lead to changes in network structure and the position of some or even all group members.^57^^,^^85^

##### Data collection part 2 (2019-2021)

Between 2019-2021, we further obtained observational data from seven independent *Pan* groups in European zoological institutes (Table S1). All the apes were housed according to the guidelines of the EAZA Ex situ Program (EEP).

### Method details

The focus of our study is on the pattern by which the apes distribute their social resources over their group members. We explicitly note that we were not interested in the apes’ time budgets, in which metrics of interactions are presented relative to the observation time. This is a common measure in primatology,^28^^,^^86^ but not the focus of this study. Hence, we only included individuals who socially interacted with at least 5 group members (to operationalize a distribution). Moreover, we focused specifically on grooming interactions, as they form an important currency in *Pan* societies – a means by which bonobos and chimpanzees not only keep themselves and their group members hygienic, but also by which they forge enduring social bonds.^37^ A grooming interaction was defined as a subject manipulating the receiver’s face and/or body surface and/or hair with its fingers or lips. Mutual grooming events were inserted as two separate interactions (one from A to B and one from B to A). Per group, we report details on their composition and the number of grooming interactions.

#### African sanctuaries

##### Chimfunshi Wildlife Orphanage Trust (Zambia)

Behavioral observations were conducted between 2018-2019 as part of a larger project aimed at assessing chimpanzee sociality over time (see^87^). Trained staff members conduct focal follows daily with an every-minute scan sampling technique in the ZooMonitor (ZM) application. The protocol comprises 10min focal follows in which 10 scan points are scored. On each scan, all instances of proximity (<1 m, including contact sitting), grooming, social play, and aggression by the focal individual are scored, including the identities of the interaction partners. Data were semi-randomly collected from the fence line, restricted by visibility. We work in a sanctuary setting in which the chimpanzees have ample space to retreat into the forest.^58^ As per sanctuary stipulations, we do not enter their enclosures ever, which prevents us from following the chimpanzees into the forest. Hence, the next best thing is to divide the fence line into different sections and start the observations randomly from these different sections, also randomizing the direction (clockwise VS counter-clockwise) in which the search for chimpanzees commences.^88^ Upon encountering a chimpanzee within eyesight, we start behavioral observations on the respective individual using established focal follow protocols (see our main text). After finishing the respective focal follow, we search for the nearest chimpanzee to start the next focal follow. Overall, if the focal follow lasted 5 minutes or less (i.e., due to visibility challenges), we discarded the focal follow. The observation efforts start at a different location each day upon which the first-seen chimpanzee is chosen as the focal. The observation efforts were distributed across the day: typically, per group, one hour was collected between 7am-11am and one hour was collected between 2:30pm and 5pm, after which the chimpanzees retreat into the forest to spend their nights there. All individuals were sampled except for dependent offspring clinging to their mothers.

The dataset for Chimfunshi comprised an average of 538 grooming interactions per group (range 411-770). Inter-observer reliability revealed good to excellent agreement between the observers (behavior: κ > 0.90; partner identity κ > 0.80).

This data collection was approved by the ethical committee of the Max Planck Institute for Evolutionary Anthropology and the data collection protocol was approved by the Chimfunshi Research Advisory Board (ref: 2014C014). Animal husbandry and research protocols complied with international standards and local guidelines on the husbandry and care for sanctuary-living animals as stipulated by the Zambia Wildlife Association (ZAWA). The study was purely observational in nature and thus did not require specific ethical approval for any changes to the daily husbandry protocols as adhered to by Chimfunshi.

##### Lola Ya Bonobo (Democratic Republic of Congo)

Data collection was conducted throughout the day, outside of feeding periods and commenced at the presence of at least one fourth of the group’s individuals to allow for observations of social interactions. Similar to the data collection procedure at Chimfunshi, we conducted 10-min focal scan follows consisting of 10 1-min scan points (see Section *4.1.1.1*). The presence of each individual as well as dyadic proximities and interactions of state behaviors were recorded (i.e., contact sit, proximity (<1m), play, groom, and sex). For the analyses of the current paper, we only used the *grooming* interactions.

The dataset comprised 1199 and 1232 grooming interactions in Group 1 and Group 2, respectively. Inter-observer reliability revealed good to excellent agreement between the observers (behavior: κ > 0.90; partner identity κ > 0.80).

The study was purely observational in nature and thus did not require specific ethical approval for any changes to the daily husbandry protocols as adhered to by Lola Ya Bonobo.

#### European zoo-settings

##### Data collection part 1 (2011-2022)

Behavioral data on social proximity (<1 meter) and grooming were collected using group scan sampling^89^ with an identical protocol in all groups. A scan was done approximately every 10-15 minutes, resulting in a mean of 626 scans per group (range 274-1103). During a scan, the behavior that each group member was performing at that moment was recorded using a standardized ethogram on a laptop with the Observer XT software (Noldus, the Netherlands). Using a group scan sampling method to collect data was possible and reliable, since our zoo-housed subjects had stable group compositions within observation periods, small group sizes (see supplemental information), and were easily visible at most times, which reduced sampling bias in our dataset to a minimum.^90^ For more details on the methods of data collection, also see.^62^

The dataset for these nine social groups comprised an average of 377 grooming interactions per group (range 195-743), which was deemed sufficient for defining reliable relationship ties.^91^ Observations were done by 11 different observers, who were subjected to rigorous training for at least 2 weeks prior to data collection and tested for inter-observer reliability by scoring the same two 10-minute bonobo videos and reached a mean of *r* = 0.85 across all observers, indicating high reliability of observations.^89^

As this is an observational study, the Royal Zoological Society of Antwerp, and the scientific advisory boards of the zoological institutions waivered the need for ethical approval.

##### Data collection part 2 (2019-2021)

The observations took place between 9am and 4pm by means of scan and all-occurrence sampling.^89^ Each observation period was 1h and we conducted maximally three periods per day. Per hour, we performed continuous group observations of all occurrences of grooming resulting in a total of 288 hours of observations with an average of 26.18 hours per group (range 21 to 42 hours). Furthermore, we conducted group-scans every 15 minutes, resulting in five scans per observation hour, and noted down which individuals remained within a distance of one meter from each other. We marked the scans during which the two individuals groomed or played with each other to obtain a measure of social proximity (<1 meter) mutually exclusively from other interactions. Using an all occurrence and group scan sampling method to collect data was possible and reliable, since our zoo-housed subjects had stable group compositions within observation periods, small group sizes, and were easily visible at most times, which reduced sampling bias in our dataset to a minimum.^90^

The dataset for these seven social groups comprised an average of 292 grooming interactions per group (range 120-459), which was deemed sufficient for defining reliable relationship ties.^91^ The observations were done by five different observers and showed interobserver agreement based on two hours of simultaneous coding per observer of a mean of *r*=0.87 for grooming.

The study was not ethically evaluated, because it was purely observational in nature and thus did not require specific ethical approval for any changes to the daily husbandry protocols as adhered to by the zoological institutes.

### Quantification and statistical analysis

#### Theoretical background and parameter estimation

Here, we describe the main results from theoretical approaches to the circle structure. In the discrete case, it is assumed that *L* is the total number of relationships in an ego-network and *σ* is the average cognitive cost of a relationship. Relationships belong to *r* different categories, each of them bearing a different cost *s*_max_ = *s*_1_ > *s*_2_ > … > *s_r_* = *s*_min_. Using a maximum entropy approach it is possible to obtain the probability that a given relationship of the ego-network belongs to category *k* as:

(Equation 1) $$p_{k}=Z_{r}^{-1}e^{-\hat{\mu }s_{k}},Z_{r}=\sum_{k=1}^{r}e^{-\hat{\mu }s_{k}}\text{,}$$

where $\hat{\mu }$ is fixed by letting *σ* be the expected cost $\sigma =E\;\left(s_{k}\right)$. Using this probability distribution we can calculate *χ_k_*, the expected number of relationships with costs larger than or equal to that of category *k* (i.e., the size of the social circles, with *k* = 1 corresponding to the innermost one), as:

(Equation 2) $$\chi_{k}=\frac{e^{k\mu }-1}{e^{r\mu }-1}\text{.}$$

Where $\mu \equiv \hat{\mu }\left(s_{\max}-s_{\min}\right)/\left(r-1\right)$. As mentioned in the main text, it can subsequently be shown that, for large values of *μ*, the scaling ratio, i.e., the size of one circle divided by the previous one, behaves approximately as:

(Equation 3) $$\frac{\chi_{k+1}}{\chi_{k}}∼e^{\mu }\;\text{if}\;\mu \to \infty \;\text{and}\;\frac{\chi_{k+1}}{\chi_{k}}∼1\;\text{if}\;\mu \to -\infty \text{.}$$

This result predicts the known regime for values of *μ* > 0, in which the circles satisfy an approximate scaling relation; in particular, for *μ* ≈1 the usual value of 3 found on empirical data is recovered. On the other hand, it also predicts a so-called ‘inverse’ regime, when *μ*< 0, in which most of the relationships are in the closest circle. This second behavior had not been described prior to the publication of Tamarit and colleagues,^2^ when it was checked against empirical data of small migrant communities, confirming its existence.

In the continuum approach, the key parameter is called *η*, and it is related to the average cost *σ* by the implicit equation:

(Equation 4) $$t\equiv \frac{s_{\max}-\sigma }{s_{\max}-s_{\min}}=\frac{e^{\eta }}{e^{\eta }-1}-\frac{1}{\eta }\text{.}$$

and thus *η* is actually a function *η*(*t*), with *t* defined in the equation above representing a normalized measure of the cost of a relationship (*t* = 0 corresponding to the highest cost and *t* = 1 to the lowest one). Once *η* is determined, the fraction of relationships with a normalized cost not larger than *t* is given by:

(Equation 5) $$\chi \left(t\right)=\frac{e^{\eta t}-1}{e^{\eta }-1}\text{.}$$

This is the curve that should fit the data. Notice that each individual will be characterized by its own value of *η*. The scaling ratio of the circles can be obtained from the asymptotic behavior, for large *η*, of the logarithmic derivative of *χ*(*t*), the fraction of links whose ‘distance’ to the individual is not larger than *t*, which turns out to be:

(Equation 6) $$\frac{\dot{\chi }\left(t\right)}{\chi \left(t\right)}=\frac{\eta e^{\eta t}}{e^{\eta t}-1}∼\eta \;\text{if}\;\eta \to \infty \;\text{and}\;\frac{\dot{\chi }\left(t\right)}{\chi \left(t\right)}=\frac{\eta e^{\eta t}}{e^{\eta t}-1}∼0\;\text{if}\;\eta \to -\infty \text{.}$$

In this approach, the separation between the two regimes, the normal and the inverted ones, also takes places at *η* = 0. Finally, to connect the two formalisms, we can use the fact that the discrete version of the left-hand side is (*χ*_*k*+1_ −*χ_k_*)/*χ_k_*Δ*t*; then, a comparison between (eqn: 3) and (eqn: 6) in the ordinary regime leads to *η*Δ*t* ≈ *e^μ^* − 1. Since Δ*t* ≈ (*r* − 1)^−1^, we obtain the equivalence:

(Equation 7) $$\eta \approx \left(r-1\right)\left(e^{\mu }-1\right)\text{.}$$

Interestingly, this result shows that the value of *μ* in the discrete model depends on the total number of layers, *r*. This fact had not been noticed in previous research because of the implicit assumption of the existence of *r* = 4 layers in the structure of ego-networks. Setting *r* = 4 in (eqn: 7) and assuming, as empirically observed, that *e^μ^* ≈ 3 (eqn: 4), we then find *η* ≈ 6.

With the above approach in mind, given a dataset of relationships with continuous weights, the scaling parameter *η* can be estimated using the maximum-likelihood method. Such an analysis leads to an expression equivalent to (eqn: 4) to connect the range of data weights to the theoretical parameters, *η* and *σ*. Thus, for an empirical dataset we can find the values of *s_max_* and *s_min_*, which are the largest/smallest possible costs an individual can invest in a relationship, respectively. Then, the value of *σ*, the total cost per item, is determined by:

(Equation 8) $$\sigma =\bar{s}=\frac{1}{L}\sum_{i=1}^{L}s_{i}$$

where *si* are the costs associated to each of the relationships, measured in the same units as *s_max_* and *s_min_*, and *L* is the total number of relationships that an individual has. Once these variables are set, the parameter *η*, that characterizes the structure of the ego-network of each individual, can be estimated solving (eqn: 4) numerically. Furthermore, an expression for the 1 − 2*δ* confidence intervals associated to the parameter *η* can be found (see^5^ for details). In what follows we choose a 95% confidence interval using *δ* = 0.025.

In summary, this paper builds on Tamarit et al.'s models,^2^^,^^5^ which theorize that relationship structures form due to finite individual capacity to invest time and effort, resulting in layered social circles with predictable scaling patterns. When parameter μ > 1, relationships expand in size but decrease in emotional closeness, aligning with observed hierarchies. For μ < 1, smaller communities show reversed layers, growing in size with greater emotional depth. A continuous model introduced parameter η, with positive or negative values indicating normal or inverted structures, respectively. Tamarit’s maximum-likelihood method estimates η from grooming data by evaluating relationship investment, providing individual-specific social structure insights.

#### Gradient boosting

Gradient boosting is a machine learning ensemble technique that combines multiple weak models to create a more robust overall model.^92^ The idea behind gradient boosting is to train a series of models gradually to minimize a differentiable loss function, e.g., log loss. The algorithm starts by training a model on the entire dataset and then computing the residuals, which are the differences between the true labels and the model’s predictions. The following model is trained to predict these residuals, and this process is repeated multiple times. Using this technique, the predictive accuracy of the ensemble improves every successive iteration because it focuses on correcting the areas in which the model is weak in the previous step. Finally, all the predictions are combined to create a more robust and accurate model. Thus, gradient boosting methods can predict linear and non-linear relationships in the data with high accuracy and low computational cost. Furthermore, the gradient boosting technique can be used for both regression and classification problems. The weak models can be decision trees, linear models, or any other model that can be trained to minimize a differentiable loss function. In particular, we used XGBoost to estimate the η parameter’s value. XGBoost is a Python library that implements gradient boosting using decision trees as base estimators.^93^

#### SHAP values

SHAP values are a method to explain the predictions of a machine learning model. They are based on the concept of Shapley values, borrowed from cooperative game theory, which measures a player’s contribution to a cooperative game.^65^ Analogously, SHAP values attribute each feature’s contribution to the final prediction of a model, calculating its expected value over all possible combinations using a technique called "sampling". This technique involves randomly generating sets of feature values that are then used to calculate the expected value of each feature’s contribution. Thus, the SHAP value for each feature is the difference between the actual and expected contributions. These values can be either positive or negative, depending on whether the variable has a positive or negative impact on the prediction. SHAP values can be used to gain insight into model decisions and to identify feature importance in a model. One of their main advantages is that they are model-agnostic, meaning they can be used to explain the predictions of any machine learning model, regardless of its underlying architecture. This is especially useful for gradient boosting methods (such as XGBoost, the one used in our analysis), which are complex and opaque, making it challenging to understand which features are driving the model’s predictions. In these cases, using SHAP values can make such models more interpretable and give a better understanding of their predictions.^65^

#### Partial dependence plots (PDPs)

To make our findings more accessible to researchers unfamiliar with machine learning techniques, we have created additional visualization plots that translate the abstract η parameter into more intuitive representations of social resource distribution patterns (see supplemental information). Specifically, we have used partial dependence plots (PDPs), which illustrate the marginal effect of a single feature on η while controlling for all other variables. PDPs are calculated by systematically varying the focal variable across its range while holding the remaining variables constant.

To produce the PDPs, we have used our XGBoost model trained on great apes’ data and, for each feature, extracted the model output while controlling other variables. Specifically, numerical variables were held constant at their median values, and categorical variables at their mode, allowing us to isolate the marginal effect of each feature (see Figures S2–S7).
