# Supplementary material for: The physics of sociality: Investigating patterns of social resource distribution among the Pan species
Source: iScience. 2025 Oct 24;28(11):113507. doi: 10.1016/j.isci.2025.113507 (PMC12682274; doi:10.1016/j.isci.2025.113507)
Supplement: Document S1. Figures S1–S7 and Tables S1 and S2 [file mmc1.pdf]

iScience, Volume *28*

Supplemental information

The physics of sociality: Investigating patterns of social resource distribution among the *Pan* species

Edwin J.C. van Leeuwen, Diego Escribano, Zanna Clay, Marcel Eens, Jean-Pascal Guéry, Daniel B.M. Haun, Stephanie Kordon, Suska Nolte, Nicky Staes, Jeroen M.G. Stevens, Jonas Torfs, José A. Cuesta, and Angel Sánchez

Figures

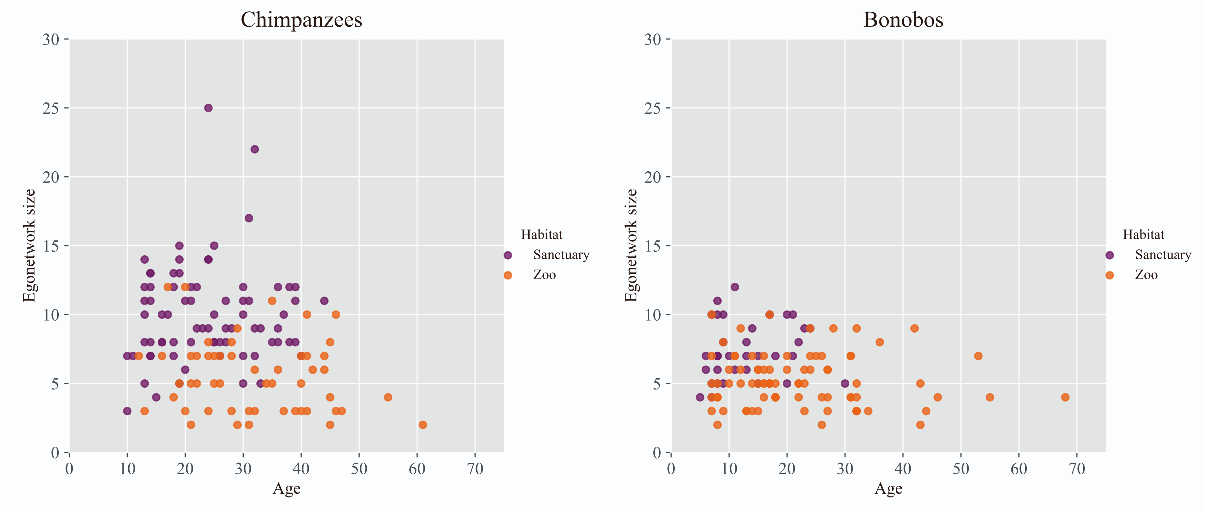

Figure S1. Scatterplot for the size of the ego-network (*y*-axis) of chimpanzees (left, n=143) and bonobos (right, n=141) plotted against age in years (*x*-axis). Symbols correspond to different environments/habitats as indicated in the legend.

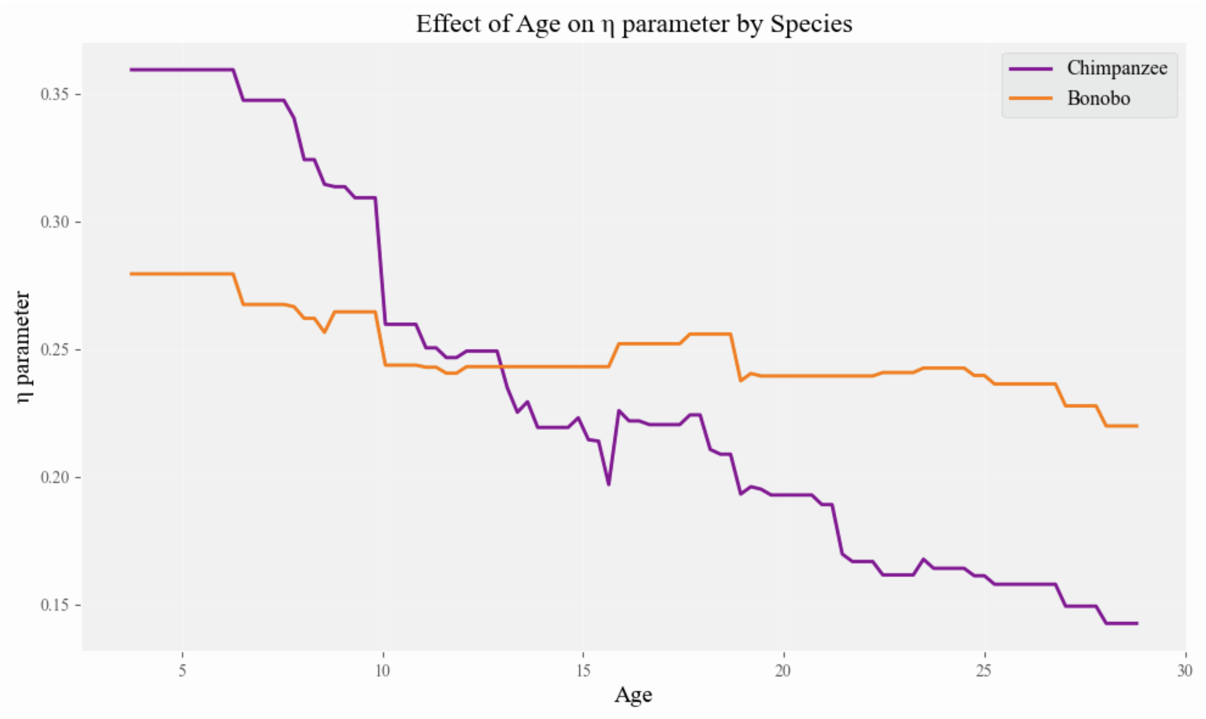

Figure S2. Partial Dependence Plot showing the marginal effect of age on η for each species separately (purple = chimpanzees; orange = bonobos).

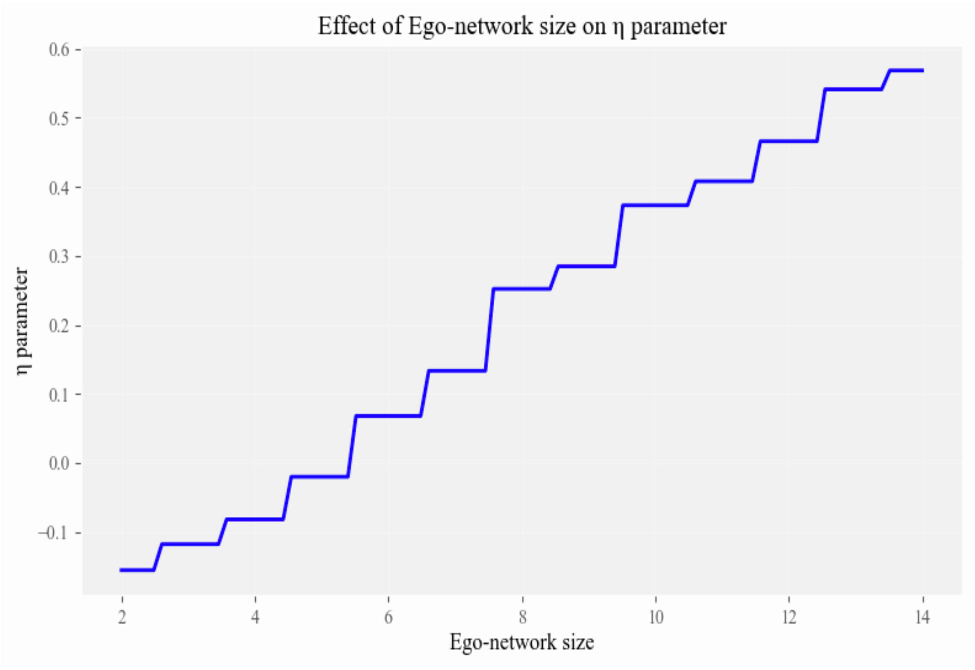

Figure S3. Partial Dependence Plot showing the marginal effect of Ego-network size on η.

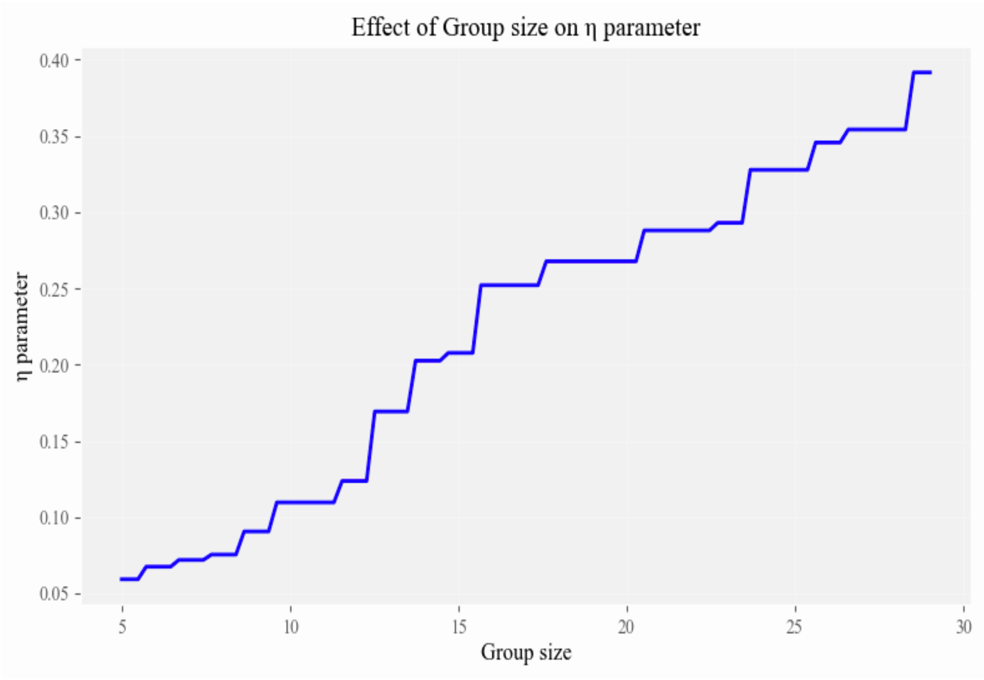

Figure S4. Partial Dependence Plot showing the marginal effect of Group size on η.

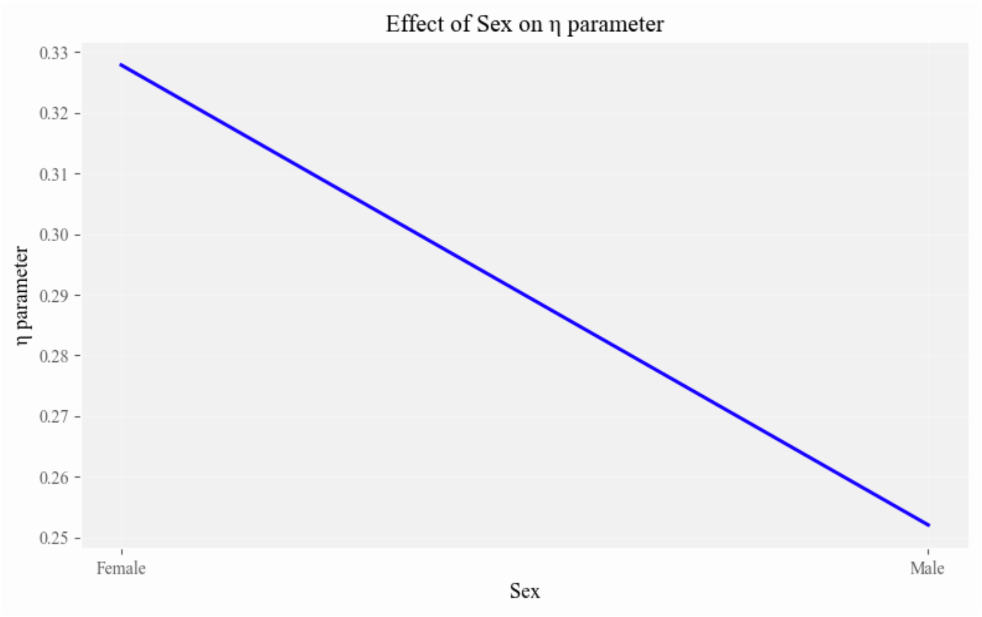

Figure S5. Partial Dependence Plot showing the marginal effect of Sex on η.

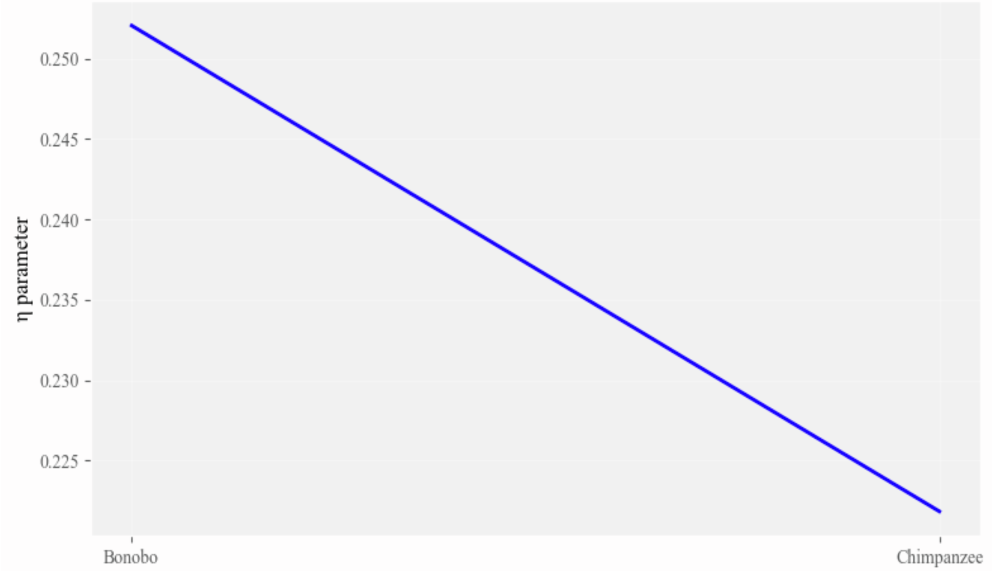

Figure S6. Partial Dependence Plot showing the marginal effect of Species on η.

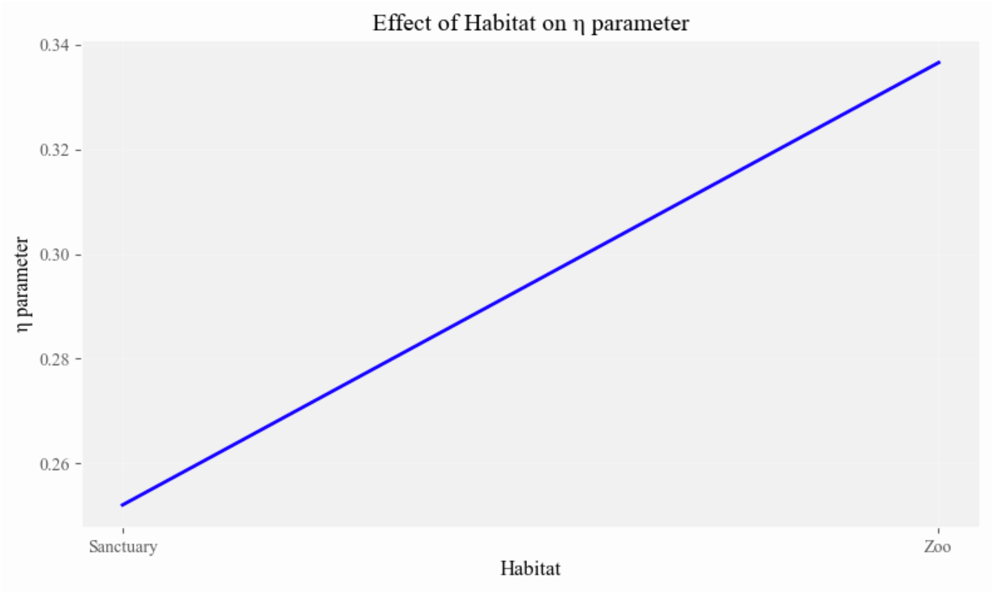

Figure S7. Partial Dependence Plot showing the marginal effect of Habitat on η.

Tables

Table S1. Subjects and subject information for all sampled apes.

Group	Individual	Species	Age*	Sex	Group size	Habitat
AP2	Bolombo	Bonobo	15	Male	6	Zoo
AP2	Hortense	Bonobo	34	Female	6	Zoo
AP2	Jill	Bonobo	27	Female	6	Zoo
AP2	Kumbuka	Bonobo	13	Female	6	Zoo
AP2	Zamba	Bonobo	14	Male	6	Zoo
AP2	Zuani	Bonobo	22	Female	6	Zoo
Apenheul	Bd	Bonobo	15	Female	6	Zoo
Apenheul	Bn	Bonobo	19	Female	6	Zoo
Apenheul	Bo	Bonobo	43	Female	6	Zoo
Apenheul	Jl	Bonobo	35	Female	6	Zoo
Apenheul	Kd	Bonobo	36	Male	6	Zoo
Apenheul	Km	Bonobo	21	Female	6	Zoo
Frankfurt_1	Bh	Bonobo	15	Female	10	Zoo
Frankfurt_1	Hi	Bonobo	20	Male	10	Zoo
Frankfurt_1	Hn	Bonobo	13	Female	10	Zoo
Frankfurt_1	Kt	Bonobo	34	Female	10	Zoo
Frankfurt_1	Mg	Bonobo	69	Female	10	Zoo
Frankfurt_1	Mx	Bonobo	19	Female	10	Zoo
Frankfurt_1	Ni	Bonobo	57	Female	10	Zoo
Frankfurt_1	No	Bonobo	14	Male	10	Zoo
Frankfurt_1	Ny.f	Bonobo	9	Female	10	Zoo
Frankfurt_1	Zo	Bonobo	23	Female	10	Zoo
Frankfurt_2	Bh	Bonobo	15	Female	11	Zoo
Frankfurt_2	Hn	Bonobo	13	Female	11	Zoo
Frankfurt_2	Kt	Bonobo	34	Female	11	Zoo
Frankfurt_2	Mg	Bonobo	69	Female	11	Zoo
Frankfurt_2	Mx	Bonobo	19	Female	11	Zoo
Frankfurt_2	Ni	Bonobo	57	Female	11	Zoo
Frankfurt_2	No	Bonobo	14	Male	11	Zoo
Frankfurt_2	Ny.f	Bonobo	9	Female	11	Zoo
Frankfurt_2	Pc	Bonobo	11	Male	11	Zoo
Frankfurt_2	Sb	Bonobo	9	Male	11	Zoo
Frankfurt_2	Zo	Bonobo	23	Female	11	Zoo
Frankfurt_4	Hannah	Bonobo	12	Female	6	Zoo
Frankfurt_4	Heri	Bonobo	18	Male	6	Zoo
Frankfurt_4	Kamiti	Bonobo	32	Female	6	Zoo

Frankfurt_4	Margrit	Bonobo	68	Female	6	Zoo
Frankfurt_4	Natalie	Bonobo	55	Female	6	Zoo
Frankfurt_4	Nayembi	Bonobo	13	Female	6	Zoo
Leipzig	Gemena	Bonobo	16	Female	7	Zoo
Leipzig	Jasongo	Bonobo	31	Male	7	Zoo
Leipzig	Kasai	Bonobo	8	Male	7	Zoo
Leipzig	Kuno	Bonobo	25	Male	7	Zoo
Leipzig	Lexi	Bonobo	22	Female	7	Zoo
Leipzig	Luiza	Bonobo	16	Female	7	Zoo
Leipzig	Yasa	Bonobo	24	Female	7	Zoo
Lola_Group1	Bandundu	Bonobo	21	Female	20	Sanctuary
Lola_Group1	Bikoro	Bonobo	5	Male	20	Sanctuary
Lola_Group1	Elikya	Bonobo	13	Female	20	Sanctuary
Lola_Group1	Katako	Bonobo	14	Female	20	Sanctuary
Lola_Group1	Kikwit	Bonobo	20	Male	20	Sanctuary
Lola_Group1	Kinsele	Bonobo	7	Male	20	Sanctuary
Lola_Group1	Kinzia	Bonobo	8	Female	20	Sanctuary
Lola_Group1	Lalia	Bonobo	6	Female	20	Sanctuary
Lola_Group1	Lopori	Bonobo	8	Male	20	Sanctuary
Lola_Group1	Lubi	Bonobo	7	Female	20	Sanctuary
Lola_Group1	Manono	Bonobo	24	Male	20	Sanctuary
Lola_Group1	Matadi	Bonobo	18	Male	20	Sanctuary
Lola_Group1	Moseka	Bonobo	6	Female	20	Sanctuary
Lola_Group1	Ndjili	Bonobo	8	Female	20	Sanctuary
Lola_Group1	Opala	Bonobo	23	Female	20	Sanctuary
Lola_Group1	Oshwe	Bonobo	11	Male	20	Sanctuary
Lola_Group1	Pole	Bonobo	13	Male	20	Sanctuary
Lola_Group1	Salonga	Bonobo	21	Female	20	Sanctuary
Lola_Group1	Semendwa	Bonobo	22	Female	20	Sanctuary
Lola_Group1	Waka	Bonobo	13	Female	20	Sanctuary
Lola_Group2	Bolingo	Bonobo	8	Male	16	Sanctuary
Lola_Group2	Boma	Bonobo	9	Female	16	Sanctuary
Lola_Group2	Bombo	Bonobo	10	Male	16	Sanctuary
Lola_Group2	Dilolo	Bonobo	17	Male	16	Sanctuary
Lola_Group2	Eleke	Bonobo	15	Male	16	Sanctuary
Lola_Group2	Garamba	Bonobo	9	Male	16	Sanctuary
Lola_Group2	Kalina	Bonobo	20	Female	16	Sanctuary
Lola_Group2	Keza	Bonobo	30	Male	16	Sanctuary
Lola_Group2	Kimya	Bonobo	8	Female	16	Sanctuary
Lola_Group2	Liyaka	Bonobo	8	Female	16	Sanctuary
Lola_Group2	Mabali	Bonobo	15	Male	16	Sanctuary
Lola_Group2	Malaïka	Bonobo	11	Female	16	Sanctuary

Lola_Group2	Minova	Bonobo	8	Female	16	Sanctuary
Lola_Group2	Moyi	Bonobo	13	Female	16	Sanctuary
Lola_Group2	Nyota	Bonobo	7	Female	16	Sanctuary
Lola_Group2	Singi	Bonobo	9	Male	16	Sanctuary
Ouwehand	Ayubu	Bonobo	11	Male	10	Zoo
Ouwehand	Azibo	Bonobo	11	Male	10	Zoo
Ouwehand	Bondo	Bonobo	31	Male	10	Zoo
Ouwehand	Eja	Bonobo	32	Female	10	Zoo
Ouwehand	Kutu	Bonobo	24	Female	10	Zoo
Ouwehand	Lingoye	Bonobo	15	Female	10	Zoo
Ouwehand	Lisala	Bonobo	42	Female	10	Zoo
Ouwehand	Luebo	Bonobo	16	Male	10	Zoo
Ouwehand	Omanga	Bonobo	14	Female	10	Zoo
Ouwehand	Visola	Bonobo	7	Female	10	Zoo
PLD	Banya	Bonobo	30	Female	9	Zoo
PLD	Busira	Bonobo	16	Female	9	Zoo
PLD	Djanoa	Bonobo	25	Female	9	Zoo
PLD	Habari	Bonobo	14	Male	9	Zoo
PLD	Hortense	Bonobo	42	Female	9	Zoo
PLD	Kianga	Bonobo	15	Female	9	Zoo
PLD	Nayoki	Bonobo	8	Female	9	Zoo
PLD	Vifijo	Bonobo	26	Male	9	Zoo
PLD	Zamba	Bonobo	22	Male	9	Zoo
PLD5	Busira	Bonobo	8	Female	6	Zoo
PLD5	Djanoa	Bonobo	17	Female	6	Zoo
PLD5	Lina	Bonobo	27	Female	6	Zoo
PLD5	Louisoko	Bonobo	14	Male	6	Zoo
PLD5	Lucuma	Bonobo	10	Male	6	Zoo
PLD5	Vifijo	Bonobo	18	Male	6	Zoo
PLD8	Banya	Bonobo	31	Female	6	Zoo
PLD8	Binti	Bonobo	26	Female	6	Zoo
PLD8	Hortense	Bonobo	43	Female	6	Zoo
PLD8	Kikongo	Bonobo	7	Male	6	Zoo
PLD8	Mokonzi	Bonobo	8	Male	6	Zoo
PLD8	Zamba	Bonobo	23	Male	6	Zoo
Stuttgart	Banbo	Bonobo	10	Female	7	Zoo
Stuttgart	Hermien	Bonobo	41	Female	7	Zoo
Stuttgart	Kianga	Bonobo	7	Female	7	Zoo
Stuttgart	Kombote	Bonobo	46	Female	7	Zoo
Stuttgart	Mobikisi	Bonobo	32	Male	7	Zoo
Stuttgart	Ximba	Bonobo	17	Female	7	Zoo
Stuttgart	Zorba	Bonobo	32	Male	7	Zoo

VDS	Daniela	Bonobo	53	Female	15	Zoo
VDS	David	Bonobo	20	Male	15	Zoo
VDS	Diwani	Bonobo	24	Male	15	Zoo
VDS	Kelele	Bonobo	17	Male	15	Zoo
VDS	Khalessi	Bonobo	9	Female	15	Zoo
VDS	Khaya	Bonobo	20	Female	15	Zoo
VDS	Lingala	Bonobo	18	Female	15	Zoo
VDS	Loto	Bonobo	12	Male	15	Zoo
VDS	Lucy	Bonobo	18	Female	15	Zoo
VDS	Moko	Bonobo	9	Male	15	Zoo
VDS	Swahili	Bonobo	7	Female	15	Zoo
VDS	Ukela	Bonobo	36	Female	15	Zoo
VDS	Ulindi	Bonobo	28	Female	15	Zoo
VDS	Yahimba	Bonobo	12	Female	15	Zoo
VDS	Yuli	Bonobo	7	Female	15	Zoo
WP	Birogu	Bonobo	23	Male	6	Zoo
WP	Busira	Bonobo	8	Female	6	Zoo
WP	Eja	Bonobo	22	Female	6	Zoo
WP	Lisala	Bonobo	32	Female	6	Zoo
WP	Lusambo	Bonobo	32	Male	6	Zoo
WP	Mato	Bonobo	44	Male	6	Zoo
Beekse Bergen1	Anne Clara	Chimpanzee	41	Female	11	Zoo
Beekse Bergen1	Dennis	Chimpanzee	35	Male	11	Zoo
Beekse Bergen1	Gert-Jan	Chimpanzee	26	Male	11	Zoo
Beekse Bergen1	Linda	Chimpanzee	36	Female	11	Zoo
Beekse Bergen1	Marijke	Chimpanzee	24	Female	11	Zoo
Beekse Bergen1	Martje	Chimpanzee	21	Female	11	Zoo
Beekse Bergen1	Michelle	Chimpanzee	25	Female	11	Zoo
Beekse Bergen1	Pepa	Chimpanzee	20	Female	11	Zoo
Beekse Bergen1	Sam	Chimpanzee	18	Male	11	Zoo
Beekse Bergen1	Stephan	Chimpanzee	13	Male	11	Zoo
Beekse Bergen1	Wakili	Chimpanzee	22	Male	11	Zoo
Beekse Bergen2	Centa	Chimpanzee	36	Female	11	Zoo
Beekse Bergen2	Daan	Chimpanzee	24	Male	11	Zoo
Beekse Bergen2	Joke	Chimpanzee	25	Female	11	Zoo
Beekse Bergen2	Jozi	Chimpanzee	9	Female	11	Zoo
Beekse Bergen2	Julian	Chimpanzee	12	Male	11	Zoo
Beekse Bergen2	Lenny	Chimpanzee	39	Female	11	Zoo
Beekse Bergen2	Lukani	Chimpanzee	31	Male	11	Zoo
Beekse Bergen2	Marlies	Chimpanzee	40	Female	11	Zoo
Beekse Bergen2	Nadine	Chimpanzee	31	Female	11	Zoo
Beekse Bergen2	Socrates	Chimpanzee	40	Male	11	Zoo

Beekse Bergen2	Wouter	Chimpanzee	21	Male	11	Zoo
Burgers Zoo	Erika	Chimpanzee	29	Female	15	Zoo
Burgers Zoo	Fons	Chimpanzee	46	Male	15	Zoo
Burgers Zoo	Gaby	Chimpanzee	37	Female	15	Zoo
Burgers Zoo	Geisha	Chimpanzee	28	Female	15	Zoo
Burgers Zoo	Ghineau	Chimpanzee	16	Male	15	Zoo
Burgers Zoo	Giambo	Chimpanzee	32	Male	15	Zoo
Burgers Zoo	Jimmie	Chimpanzee	61	Female	15	Zoo
Burgers Zoo	Jing	Chimpanzee	40	Male	15	Zoo
Burgers Zoo	Moni	Chimpanzee	32	Female	15	Zoo
Burgers Zoo	Moniek	Chimpanzee	44	Female	15	Zoo
Burgers Zoo	Morami	Chimpanzee	34	Female	15	Zoo
Burgers Zoo	Raimee	Chimpanzee	22	Female	15	Zoo
Burgers Zoo	Roosje	Chimpanzee	42	Female	15	Zoo
Burgers Zoo	Tesua	Chimpanzee	35	Female	15	Zoo
Burgers Zoo	Tushi	Chimpanzee	29	Female	15	Zoo
CWOT_Group1	BJ	Chimpanzee	13	Female	22	Sanctuary
CWOT_Group1	Bob	Chimpanzee	20	Male	22	Sanctuary
CWOT_Group1	Booboo	Chimpanzee	39	Male	22	Sanctuary
CWOT_Group1	Brenda	Chimpanzee	25	Female	22	Sanctuary
CWOT_Group1	Chrissie	Chimpanzee	14	Female	22	Sanctuary
CWOT_Group1	Genny	Chimpanzee	24	Female	22	Sanctuary
CWOT_Group1	Gerard	Chimpanzee	19	Male	22	Sanctuary
CWOT_Group1	Girly	Chimpanzee	39	Female	22	Sanctuary
CWOT_Group1	Gonzaga	Chimpanzee	13	Female	22	Sanctuary
CWOT_Group1	Ilse	Chimpanzee	18	Female	22	Sanctuary
CWOT_Group1	Ingrid	Chimpanzee	30	Female	22	Sanctuary
CWOT_Group1	Innocentia	Chimpanzee	14	Female	22	Sanctuary
CWOT_Group1	Irene	Chimpanzee	9	Female	22	Sanctuary
CWOT_Group1	Pal	Chimpanzee	40	Male	22	Sanctuary
CWOT_Group1	Rachel	Chimpanzee	8	Female	22	Sanctuary
CWOT_Group1	Regina	Chimpanzee	14	Female	22	Sanctuary
CWOT_Group1	Renate	Chimpanzee	24	Female	22	Sanctuary
CWOT_Group1	Richard	Chimpanzee	6	Male	22	Sanctuary
CWOT_Group1	Rita	Chimpanzee	38	Female	22	Sanctuary
CWOT_Group1	Rusty	Chimpanzee	14	Male	22	Sanctuary
CWOT_Group1	Tara	Chimpanzee	38	Male	22	Sanctuary
CWOT_Group1	Tobar	Chimpanzee	39	Male	22	Sanctuary
CWOT_Group2	Carol	Chimpanzee	24	Female	40	Sanctuary
CWOT_Group2	Charity	Chimpanzee	13	Female	40	Sanctuary
CWOT_Group2	Claire	Chimpanzee	18	Female	40	Sanctuary
CWOT_Group2	Coco	Chimpanzee	36	Female	40	Sanctuary

CWOT_Group2	Daisey	Chimpanzee	16	Female	40	Sanctuary
CWOT_Group2	Danny	Chimpanzee	9	Male	40	Sanctuary
CWOT_Group2	David	Chimpanzee	19	Male	40	Sanctuary
CWOT_Group2	Debbie	Chimpanzee	19	Female	40	Sanctuary
CWOT_Group2	Diana	Chimpanzee	30	Female	40	Sanctuary
CWOT_Group2	Dizzy	Chimpanzee	13	Female	40	Sanctuary
CWOT_Group2	Dolly	Chimpanzee	24	Female	40	Sanctuary
CWOT_Group2	Donna	Chimpanzee	37	Female	40	Sanctuary
CWOT_Group2	Dora	Chimpanzee	32	Female	40	Sanctuary
CWOT_Group2	Doug	Chimpanzee	18	Male	40	Sanctuary
CWOT_Group2	Little Jacky	Chimpanzee	9	Male	40	Sanctuary
CWOT_Group2	Little Jane	Chimpanzee	36	Female	40	Sanctuary
CWOT_Group2	Little Jenkins	Chimpanzee	14	Female	40	Sanctuary
CWOT_Group2	Little Jones	Chimpanzee	10	Female	40	Sanctuary
CWOT_Group2	Little Judy	Chimpanzee	25	Female	40	Sanctuary
CWOT_Group2	Long John	Chimpanzee	14	Male	40	Sanctuary
CWOT_Group2	Maggie	Chimpanzee	35	Female	40	Sanctuary
CWOT_Group2	Mary	Chimpanzee	15	Female	40	Sanctuary
CWOT_Group2	Masya	Chimpanzee	30	Female	40	Sanctuary
CWOT_Group2	Max	Chimpanzee	14	Male	40	Sanctuary
CWOT_Group2	Maxine	Chimpanzee	19	Female	40	Sanctuary
CWOT_Group2	May	Chimpanzee	8	Female	40	Sanctuary
CWOT_Group2	Mikey	Chimpanzee	33	Male	40	Sanctuary
CWOT_Group2	Misha	Chimpanzee	33	Female	40	Sanctuary
CWOT_Group2	Moyo	Chimpanzee	13	Male	40	Sanctuary
CWOT_Group2	Nikkie	Chimpanzee	23	Female	40	Sanctuary
CWOT_Group2	Nina	Chimpanzee	18	Female	40	Sanctuary
CWOT_Group2	Noel	Chimpanzee	44	Female	40	Sanctuary
CWOT_Group2	Pan	Chimpanzee	32	Male	40	Sanctuary
CWOT_Group2	Pippa	Chimpanzee	32	Female	40	Sanctuary
CWOT_Group2	Tess	Chimpanzee	22	Female	40	Sanctuary
CWOT_Group2	Tilly	Chimpanzee	20	Female	40	Sanctuary
CWOT_Group2	Trixie	Chimpanzee	31	Female	40	Sanctuary
CWOT_Group2	Violet	Chimpanzee	30	Female	40	Sanctuary
CWOT_Group2	Vis	Chimpanzee	17	Male	40	Sanctuary
CWOT_Group2	Zsabu	Chimpanzee	31	Male	40	Sanctuary
CWOT_Group3	Barbie	Chimpanzee	25	Female	10	Sanctuary
CWOT_Group3	Brent	Chimpanzee	7	Female	10	Sanctuary
CWOT_Group3	Brian	Chimpanzee	27	Male	10	Sanctuary
CWOT_Group3	Bruce	Chimpanzee	11	Male	10	Sanctuary
CWOT_Group3	Buffy	Chimpanzee	36	Female	10	Sanctuary
CWOT_Group3	Bussie	Chimpanzee	16	Male	10	Sanctuary

CWOT_Group3	Clement	Chimpanzee	28	Male	10	Sanctuary
CWOT_Group3	E.T.	Chimpanzee	26	Female	10	Sanctuary
CWOT_Group3	Lods	Chimpanzee	10	Female	10	Sanctuary
CWOT_Group3	Roxy	Chimpanzee	26	Female	10	Sanctuary
CWOT_Group4	Bobby	Chimpanzee	27	Male	14	Sanctuary
CWOT_Group4	Commander	Chimpanzee	21	Male	14	Sanctuary
CWOT_Group4	Grace	Chimpanzee	6	Female	14	Sanctuary
CWOT_Group4	Jack	Chimpanzee	13	Male	14	Sanctuary
CWOT_Group4	Jewel	Chimpanzee	7	Male	14	Sanctuary
CWOT_Group4	Kambo	Chimpanzee	25	Female	14	Sanctuary
CWOT_Group4	Kathy	Chimpanzee	22	Female	14	Sanctuary
CWOT_Group4	Ken	Chimpanzee	9	Male	14	Sanctuary
CWOT_Group4	Kit	Chimpanzee	16	Male	14	Sanctuary
CWOT_Group4	Leila	Chimpanzee	12	Female	14	Sanctuary
CWOT_Group4	Miracle	Chimpanzee	21	Female	14	Sanctuary
CWOT_Group4	Nicky	Chimpanzee	30	Male	14	Sanctuary
CWOT_Group4	Sinkie	Chimpanzee	27	Male	14	Sanctuary
CWOT_Group4	Val	Chimpanzee	21	Male	14	Sanctuary
Leipzig_a	Bambari	Chimpanzee	21	Female	14	Zoo
Leipzig_a	Corry	Chimpanzee	45	Female	14	Zoo
Leipzig_a	Dorien	Chimpanzee	41	Female	14	Zoo
Leipzig_a	Fraukje	Chimpanzee	45	Female	14	Zoo
Leipzig_a	Frodo	Chimpanzee	28	Male	14	Zoo
Leipzig_a	Lobo	Chimpanzee	17	Male	14	Zoo
Leipzig_a	Lome	Chimpanzee	20	Male	14	Zoo
Leipzig_a	Maja	Chimpanzee	35	Female	14	Zoo
Leipzig_a	Natascha	Chimpanzee	41	Female	14	Zoo
Leipzig_a	Riet	Chimpanzee	44	Female	14	Zoo
Leipzig_a	Robert	Chimpanzee	46	Male	14	Zoo
Leipzig_a	Sandra	Chimpanzee	28	Female	14	Zoo
Leipzig_a	Swela	Chimpanzee	26	Female	14	Zoo
Leipzig_a	Tai	Chimpanzee	19	Female	14	Zoo
Leipzig_b	Alex	Chimpanzee	20	Male	6	Zoo
Leipzig_b	Daza	Chimpanzee	45	Female	6	Zoo
Leipzig_b	Frederike	Chimpanzee	47	Female	6	Zoo
Leipzig_b	Hope	Chimpanzee	31	Female	6	Zoo
Leipzig_b	Jeudi	Chimpanzee	55	Female	6	Zoo
Leipzig_b	Zira	Chimpanzee	24	Female	6	Zoo

*at the start of the study

Table S2. Correlation matrix between the different independent variables in our analyses.

	Age	Group size	Ego-network size	Sex (Male)	Species (Chimpanzee)	Habitat (Zoo)
**Age**	1.00	-0.08	0.00	-0.19	0.25	0.29
**Group size**	-0.08	1.00	0.41	-0.08	0.47	-0.63
**Ego-network size**	0.00	0.41	1.00	-0.02	0.27	-0.48
**Sex (Male)**	-0.19	-0.08	-0.02	1.00	-0.03	-0.06
**Species (Chimpanzee)**	0.25	0.47	0.27	-0.03	1.00	-0.33
**Habitat (Zoo)**	0.29	-0.63	-0.48	-0.06	-0.33	1.00
